# Self-assessment of attitudes towards conditions to provide safe abortion among new medical graduates in Thailand, 2018: an application of cross-sectional survey with factor analysis

**DOI:** 10.1186/s12905-021-01412-3

**Published:** 2021-07-27

**Authors:** Nithiwat Saengruang, Nisachol Cetthakrikul, Anond Kulthanmanusorn, Somtanuek Chotchoungchatchai, Nareerut Pudpong, Rapeepong Suphanchaimat

**Affiliations:** 1 International Health Policy Program, Ministry of Public Health, Nonthaburi, 11000 Thailand; 2 Bo Kluea Hospital, Nan, 55220 Thailand; 3 Division of Epidemiology, Department of Disease Control, Nonthaburi, 11000 Thailand

**Keywords:** Safe abortion, Medical graduates, Attitudes, Reproductive health, Factor analysis, Thailand

## Abstract

**Background:**

Unsafe abortion is one of the major public health problems in Thailand. Although the penal code of Thailand and the Thai Medical Council permit doctors to perform safe abortion in certain conditions, little is known about the attitudes that new medical doctors have towards abortion. The objectives of this article are to explore the attitudes towards abortion in certain conditions among new medical graduates and to identify factors related to those attitudes.

**Methods:**

A cross-sectional survey was conducted in 2018 among 2017 medical graduates who attended the annual workplace selection forum. The participants came from the two main tracks of admission to Thai medical schools: normal track and special track physicians, namely, the Collaborative Project to Increase Production of Rural Doctors (CPIRD). Of these 2017 graduates, 926 returned the questionnaire with complete information. Descriptive analysis, factor analysis, and multi-variable regression analysis were performed.

**Results:**

We found that most physicians agreed to perform abortions in the context of life-threatening conditions for mothers and children, but not under conditions directly related to physical health (such as pregnancy with socioeconomic problems or pregnancy in adolescents). CPIRD doctors were less amenable than normal track doctors in providing abortions if the reason for the termination of pregnancy was related to socioeconomic problems.

**Conclusion:**

The study suggests that a proactive campaign for new medical graduates to raise awareness and mutual understanding of abortion services should be exercised. The CPIRD curricula relating to safe abortion should enhance the capacity of medical graduates to deal with pregnant women who face not only a physical health-related problem, but also socioeconomic difficulties and well-being as a whole.

**Supplementary Information:**

The online version contains supplementary material available at 10.1186/s12905-021-01412-3.

## Background

Unsafe abortion is a serious public health problem that compromises women's health around the world. Globally, it is estimated that each year, about 25.1 million women have unsafe abortions and approximately 193,000 women die as a result [1, 2]. These deaths are preventable by having safe abortion under competent healthcare workers with appropriate skills as recommended by the World Health Organization (WHO) [3]. Countries with restrictive abortion policies usually face a high prevalence of unsafe abortion, and maternal mortality rates in these countries are about three to four times as high as the rates in countries with relatively liberal abortion policies [4].

In Thailand, public regulations for abortion have gradually developed since 1956. The Government endorsed the offense against life and body, including abortion, as part of the Penal Code, B.E. 2499 (A.D.1956) and this is still enforced until present. The law stipulates that abortion is not permitted, except when it is provided by a medical practitioner with a woman’s consent under two restricted conditions: (1) to protect health of a woman, and (2) to help pregnant women from criminal offences such as rape, seduction of a girls under 15 years, and deceitful misconduct for sexual activities [5]. However, the term ‘woman’s health’ is not clearly defined in the law. This raises the question among academia and physicians: to what extent does this law encompass woman’s health? In 2005, the Medical Council of Thailand (TMC) tried to address this problem by expanding the scope of woman’s health to physical and mental health including stress and depression [6].

In 2016, the ‘Act for Prevention and Solution of Adolescent’s Pregnancy Problem B.E. 2559 (2016) was promulgated. The Act implies that abortion is a reproductive health service and an adolescent can make his/her own decision without a permission from the parents (if he/she is provided with proper and adequate information about the rights to abortion) [7]. In other words, this legal instrument creates room for a healthcare provider to be free from the criminal offence specified in the existing penal code. Also, in 2017, safe abortion became an item in the benefit package of the Thai main public health insurance scheme [8]. This means a woman undergoing abortion will have no out-of-pocket payment at point of care. The public health facilities providing abortion to a woman can be reimbursed the cost of treatment from the central purchaser (in this case, the National Health Security Office) based on a set fee-schedule. Further, the new regulation of the TMC stipulates that all new medical graduates must be able to perform manual vacuum aspiration (MVA), whereas the prior regulation specified sharp curettage as a minimal requirement [9].

All of these mechanisms intend to facilitate the access to safe abortion services for women since there have been a report in the media from time to time about morbidity and mortality occurring in women undergoing abortion in (unauthorized) private clinics. A report by Chaturachinda suggested that approximately 30,000 abortions took place in the Thai public facilities each year, yet most abortions are carried out in private facilities or in unmarked abortion clinics or by self-induction—perhaps more than four-fold outnumbering the services in public data.

In the wider public’s view, abortion is a sensitive issue as it is perceived as sin or demerit [10]. The discretion of the medical practitioners who perform abortions plays a pivotal role in determining the accessibility of safe abortion for women. Medical doctors’ negative attitudes towards abortion may lead them to deny abortions which can ultimately compromise the health of woman in need [11]. A study in Chile presented that medical and midwifery students from secular universities have more positive attitude towards abortion than students from religiously-affiliated universities [12]. Another study in Nepal found that a lack of training and drug supplies as well as insufficient reasons for abortion were the main cause of denied abortion [13].

Apart from the denial of abortion among doctors, gestational age, health conditions and logistic barriers are also the cause of denied abortion among clients. There was a study in Tunisia, revealing, a except for medical necessity, women decided to turn away from abortion [14]. Other studies showed that late abortion may resulted from waiting to know sex of fetal or financial problem and unawareness about legal abortion services [14–16].

To date, there are limited studies on the attitudes of medical practitioners towards abortion in Thailand. The most recent study, which applied a nationwide survey on the attitudes to abortion among Thai physicians, was conducted in 2003 and no information has been updated since then. In that study, most sampled physicians (90.3%) concerned about unsafe abortion, and more than half of the physicians viewed that women should be able access safe abortion without offence, if the pregnancy was involved with rape or incest or certain health conditions (such as disorders of fetus, HIV/AIDS, and mental problems). [17]. Another study was carried out in. The study found that proper training in medical schools had positive impact on the attitudes to incorporate abortion practice among new medical graduates [18].

After 2017, the medical graduating system in Thailand has changed massively. In the past, about 1,000 new medical students graduated each year and almost all of them were recruited through the national entrance exam, the so-called, ‘normal track students’. However, in the past few years, the production capacity of medical schools has risen enormously. At present, more than 2,500 new medical students graduate each year. In addition, the normal track system is not the sole recruitment channel anymore as the special admission track has been introduced, namely, ‘the Collaborative Project to Increase Production of Rural Doctors (CPIRD)’ [19].

For the normal track, medical students are recruited from the national entrance examination. All grade-12 high school students are required to sit in the exam before being recruited. Upon graduation, they can choose to work anywhere in the public sector. For the CPIRD track, medical students are recruited at a provincial level without the need to sit in the national entrance exam as the exam is done at the provincial level. The difference from the normal track is that, upon graduation, CPIRD graduates are obliged to work in the province in which their residential address is located. During the study years, medical students of both tracks need to study basic sciences during their first three years, and another three years for clinical practices. The students in the normal track are generally trained in super-tertiary-care university-based medical schools, while those in the CPIRD track are mostly trained in regional and provincial hospitals. The faculties of regional and provincial hospitals for the CPIRD track are mostly in-service physicians of the Ministry of Public Health (MOPH), while the faculties of the normal track are mostly specialized medical officers in university hospitals. The difference in training contexts means there is a possibility of having difference attitudes towards the care of patients, including reproductive health, between graduates from the normal track and from the CPIRD track.

Therefore, the objectives of this article are to: (1) explore the overall attitudes towards safe abortion among new medical graduates in Thailand; (2) compare the attitude of conditions for performing safe abortion between the normal track graduates and the CPIRD graduates; and (3) identify factors that potentially determine the degree of acceptance to perform safe abortion in various conditions among new medical graduates.

## Methods

### Study design, population, and sample size

We performed a cross-sectional survey among new medical graduates in 2018. Participants were new medical graduates who attended the annual orientation forum during 16–17 May 2018. The forum is organized by the MOPH every year and serves as an official platform to provide necessary information about workplace selection for new graduates. After attending the forum, the graduates are obliged to select the workplace in public sector. In 2018 there were 2662 new graduates all over the country. These graduates were not yet trained as a specialist. Of these, there were a total of 2017 (75.7%) medical graduates attending the forum. We did not calculate the sample size as we intended to invite all graduates in the forum to join the survey. Graduates who did not attend the forum were those who declined to work in public facilities and were subject to fines. Those missing the forum were excluded from the survey. All of the forum attendees were invited to participate in the survey by the staff of the forum. The QR code to access the online questionnaire was provided to them on flyers, and the staff of the forum informed the participants about the survey process via audio-broadcasting. Information about the project and consent forms were also disseminated, along with the online questionnaire. The participants were asked to sign the online consent form before answering the questions.

### Questionnaire design and distribution

The questionnaire was developed by employing the 2003 survey of the Department of Health (DOH), MOPH, Thailand. Additional information derived from “the Abortion Policies and Reproductive Health around the World” of the United Nations [4, 17] also served as inputs for questionnaire development. After the questionnaire was drafted, we arranged a few rounds of consultative meetings with experts, obstetrics and gynecologists (OB-GYN doctors), reproductive-health academics, government officials, general practitioners (GP) and nurses at district hospitals, and representatives from non-government organizations (NGO), numbering about 8–10 participants in each round. Each meeting lasted approximately two hours. The aim of the meetings was to assess the questionnaire validity and to ensure that the questions used in the survey were relevant to policy interests.

The questionnaire comprised two main parts: (1) demographic data and education profiles of the respondents; and (2) self-assessment of medical graduates’ attitude towards conditions under which to perform safe abortion.

In the first part, the questionnaire included certain variables, namely, sex (male vs. female), age (years using the figure of 25 years, the mean age of new medical graduates, as a cut-off), region of domicile where the respondents were brought up during the first fifteen years of life, mode of admission (normal track v CPIRD), confidence in providing antenatal care (ANC) using self-rated Likert scale from 1 to 4 (1 = least confidence and 4 = most confidence), and intention to continue further training to be an OB-GYN doctor (yes vs. no). These variables were selected by a discussion among the research team members and the consultation with a few OB-GYN and public-health experts mad most of these variables had been ever mentioned in prior literature [20]. Note that the actual questionnaire asked the types of specialty in which the graduates planned to be involved as their career path grew. However, in this study, we focused on OB-GYN specialty only. The full list of specialties and the number of respondents intending to join each specialty are presented in Additional file 1.

In the second part, respondents were asked about their views towards conditions under which to provide safe abortion services. Fourteen conditions were set up, comprising: (1) to save a woman’s life; (2) to preserve a woman's mental health; (3) to preserve a woman's physical health; (4) a pregnant woman living with HIV/AIDS; (5) pregnancy due to failed contraception; (6) pregnancy after rape; (7) pregnancy in incestuous relationships; (8) pregnancy in girls aged below 15 years; (9) pregnancy in teenagers aged 15–20 years; (10) a pregnant woman suffering from domestic violence; (11) non-marital pregnancy; (12) pregnancy with socioeconomic problems; (13) pregnancy with fetal impairment; and (14) abortion upon reasonable request after adequate counseling. A self-rated Likert scale was used (1 = totally disagree and 5 = totally agree). The full text describing each condition (both in original Thai and English translated version) is displayed in Additional file 2.

The time used to fill in the questionnaire was about ten minutes on average. The completed data were sent online to the researchers’ personal computer with password protection. All individual identity data were automatically encrypted to protect confidentiality.

### Data analysis

The analysis plan consisted of three steps. First, the demographic data and self-rated attitudes were analyzed by descriptive statistics. Then, t-test and Chi-square test were employed to explore the difference of characteristics and attitudes between normal track and CPIRD graduates.

Second, factor analysis was performed in order to construct a few composite items that provided better interpretation (rather than to reduce a shorter form of questionnaire). In this step, we began with checking if the raw data were suitable for factor analysis by Bartlett sphericity test. Then, the 14 set-up conditions were grouped together by a principal component factor (PCF) technique [21]. Latent factors with eigenvalue equal to or greater than 1, or those showing a sharp levelling off in scree plot (the ‘elbow’ of the graph) were retained [22, 23]. Then, varimax rotation was applied to group conditions that were similar to each other together. After that, factor scores were generated by Bartlett’s formula. With this technique, the mean score of the factors would approximately equal zero. Factors with the score above zero indicated a greater degree of acceptance to perform safe abortion in that condition while those showing the score below zero indicated an opposite direction of acceptance.

Third, the relationship between mode of admission and attitudes to perform safe abortion in various conditions was determined. The dependent variable in this step was the score of each composite factor acquired from the factor analysis. The independent variables were sex, age, regional domicile during the early years of life, mode of admission, confidence in providing ANC, and intention to become an OB-GYN doctor.

We then commenced with univariable analysis (t-test) on each demographic variable, one by one. Then we performed multivariable regression analysis by including all independent variables in the model all at once. Note that, for better interpretation of the result, the confidence in providing ANC was re-classified as low confidence (rated 1–2) and high confidence (rated, 3–4). Furthermore, this study used ‘region’ variable as a proxy for ‘religion’. A reason for this was that, in the south of Thailand, the percentage of population who was Muslim was 28%, whereas there was less than 10% of population was Muslim in other regions [24]. Presumably, graduates from the southern region were Muslim, while those from the other regions were not. All analyses were performed by using STATA version 14.2 (serial number: 401406358220).

**Fig. 1:**
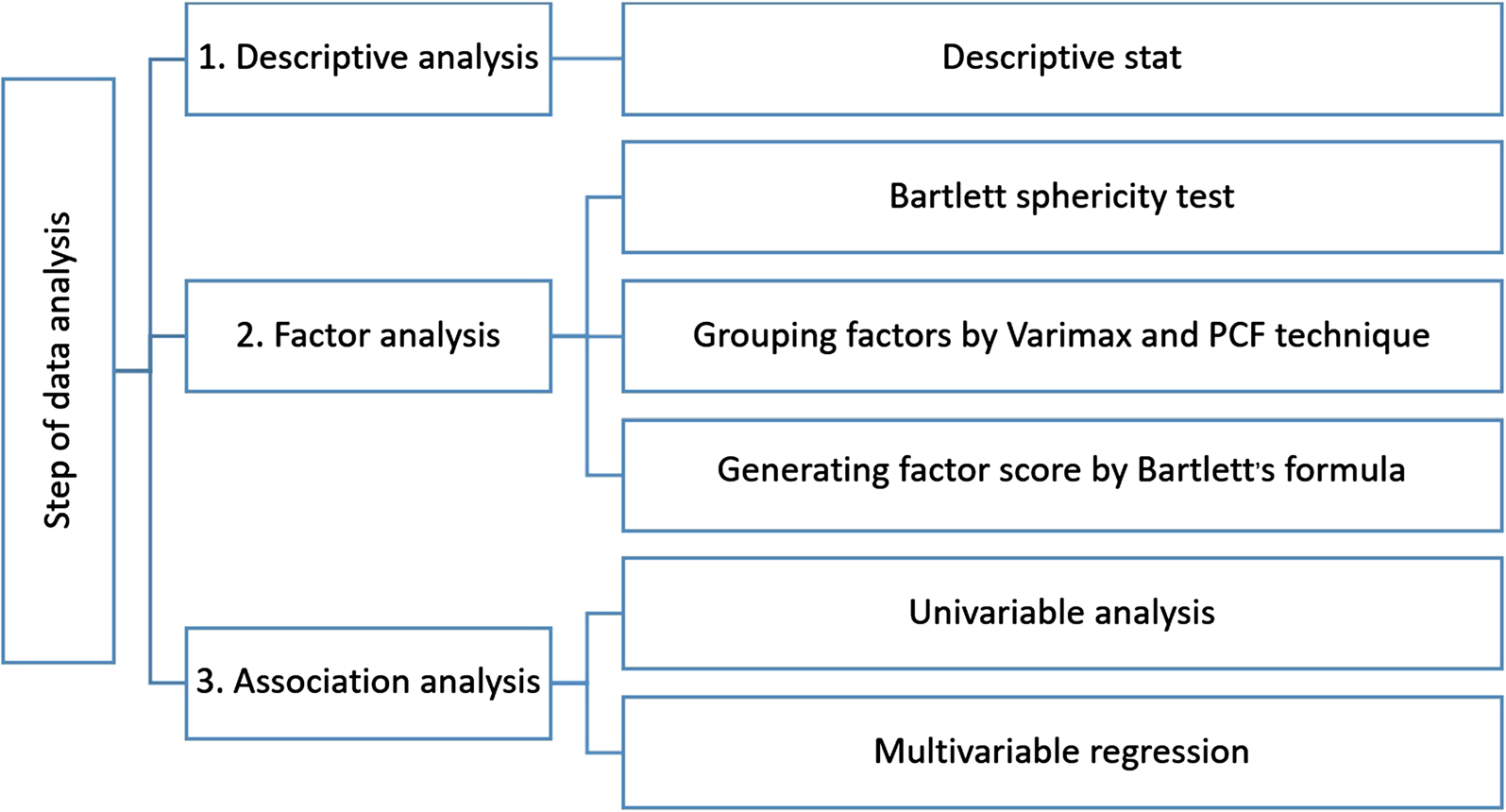
Summary of the analysis steps

The overview of the analysis steps is summarized in Fig. 1.

## Results

### Descriptive statistics and attitudes towards conditions for performing safe abortion

Of the 2017 medical graduates attending the survey, 926 returned the questionnaires with complete information (response rate = 45.9%). Most respondents were females (58.2%), with a mean age of 24.5 years and a median age of 24.0 years. About half of the participants (n = 489 or 53.0%) were CPIRD graduates. Age distribution and intention to become an OB-GYN doctor exhibited no significant difference between normal track and CPIRD graduates. The proportion of female respondents were higher in the CPIRD group (63.4%) compared with the normal track group (52.4%). Moreover, a greater proportion of those reporting a high confidence in ANC was observed in CPIRD graduates (71.2% for normal track v 7.3% for CPIRD). In addition, most normal track was born and grew up in the central region (72.0%), while most CPIRD graduates originated from the northeastern region (Table 1).

**Table 1:** Profile of respondents

Characteristics	Overall (n = 926)	Normal track (n = 437)	CPIRD (n = 489)	P-value
Mean age (standard deviation)—years	24.5 (1.8)	24.3 (1.3)	24.7 (2.2)	0.002
Age group, n (%)				0.306
< 25 years	709 (76.6)	328 (75.1)	381 (77.9)	
> = 25 years	217 (23.4)	109 (24.9)	108 (22.1)	
Sex, n (%)				0.001
Female	539 (58.2)	229 (52.4)	310 (63.4)	
Male	387 (41.8)	208 (47.6)	179 (36.6)	
Region, n (%)				< 0.001
North	214 (23.1)	85 (39.7)	129 (60.3)	
Central	329 (35.5)	237 (72.0)	92 (28.0)	
Northeast	213 (23.0)	44 (20.7)	169 (79.3)	
South	170 (18.4)	71 (41.8)	99 (58.2)	
Confidence in ANC competency, n (%)				0.033
Low confidence	237 (25.6)	126 (28.8)	111 (22.7)	
High confidence	689 (74.4)	311 (71.2)	378 (77.3)	
Intention to be an OB-GYN doctor, n (%)				0.398
Yes	49 (5.3)	26 (5.9)	23 (4.7)	
No	877 (94.7)	411 (94.1)	466 (95.3)	

*CPIRD* the Collaborative Project to Increase Production of Rural Doctors, *OB-GYN doctor*, obstetrics and gynecologists

Table 2 reports an average score of respondent’s attitudes towards safe abortion in 14 pregnancy conditions. In a macro-view, four out of 14 conditions showed a mean score of more than four, that is, to save a woman’s life (4.7), pregnancy after rape (4.5), fetal impairment (4.5), and to preserve a woman's physical health (4.3). In contrast, the lowest mean score was found in non-marital pregnancy (1.7), followed by pregnancy in women aged 15–20 years (1.9), and below 15 years (2.1). The normal track group seemed to have more positive attitudes than the CPIRD group in most of the conditions. The largest difference of attitudes between the normal track and the CPIRD was found in the item, pregnancy aged below 15 years (normal track = 2.3 vs. CPIRD = −1.9). Most participants showed a positive attitude towards abortion services upon request after adequate counselling, with a mean score of 3.2 in both CPIRD and normal track graduates.

**Table 2:** Mean score (standard deviation) of attitudes toward safe abortion in various conditions

Conditions	Overall respondents	Normal track	CPIRD
1. To save a woman’s life	4.7 (0.6)	4.7 (0.5)	4.6 (0.7)
2. To preserve a woman's mental health	3.9 (0.9)	4.0 (0.8)	3.8 (1.0)
3. To preserve a woman's physical health	4.3 (0.7)	4.4 (0.7)	4.3 (0.7)
4. Pregnant woman living with HIV/AIDS infection	2.2 (1.2)	2.2 (1.2)	2.1 (1.1)
5. Failed contraception	2.2 (1.2)	2.3 (1.2)	2.0 (1.1)
6. Pregnancy after rape	4.5 (0.7)	4.5 (0.7)	4.4 (0.8)
7. Pregnancy in incestuous relationship	2.7 (1.1)	2.8 (1.1)	2.6 (1.1)
8. Pregnancy in women aged below 15 years	2.1 (1.2)	2.3 (1.2)	1.9 (1.1)
9. Pregnancy in women aged 15–20 years	1.9 (1.0)	2.1 (1.1)	1.8 (0.9)
10. Suffering from domestic violence	2.4 (1.1)	2.6 (1.1)	2.3 (1.0)
11. Non-marital pregnancy	1.7 (0.9)	1.8 (1.0)	1.6 (0.8)
12. Socioeconomic problems	2.2 (1.1)	2.4 (1.2)	2.1 (1.1)
13. Fetal impairment	4.5 (0.8)	4.5 (0.7)	4.6 (0.9)
14. Abortion upon reasonable request after adequate counseling	3.2 (1.2)	3.2 (1.2)	3.2 (1.1)

*CPIRD* the Collaborative Project to Increase Production of Rural Doctors

### Factor analysis

**Fig. 2:**
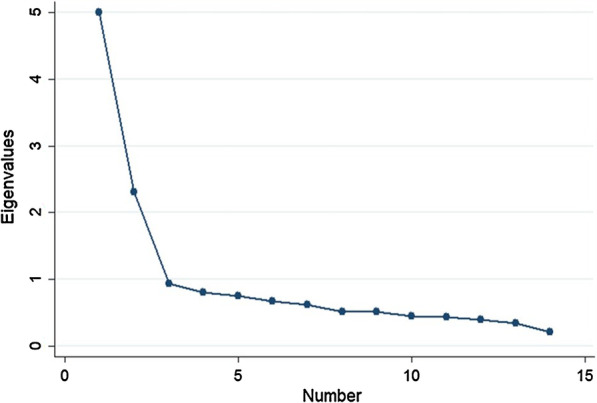
Scree plot of the fourteen set-up conditions

The Bartlett sphericity test showed a P-value of less than 0.001, suggesting that the 14 questions on the conditions for safe abortion were suitable for factor analysis. The PCF technique produced two factors with an eigenvalue of more than one (Table 3), but the scree plot suggested that the acute angle of the graph presented after the presentation of the third factor. Hence, we decided to keep three latent factors (Fig. 2).

**Table 3:** Eigenvalues of all 14 conditions for safe abortion based on factor analysis

Factor	Eigenvalue	Proportion of total variance	Sum of variance
Factor1	4.99744	0.35700	0.35700
Factor2	2.31033	0.16500	0.52200
Factor3	0.93370	0.06670	0.58870
Factor4	0.80770	0.05770	0.64640
Factor5	0.75185	0.05370	0.70010
Factor6	0.67862	0.04850	0.74850
Factor7	0.62897	0.04490	0.79350
Factor8	0.52292	0.03740	0.83080
Factor9	0.51755	0.03700	0.86780
Factor10	0.45644	0.03260	0.90040
Factor11	0.44201	0.03160	0.93200
Factor12	0.39645	0.02830	0.96030
Factor13	0.34078	0.02430	0.98460
Factor14	0.21523	0.01540	1.00000

Then, a varimax rotation was performed. Table 4 presents a factor-loading matrix after rotation. For more convenience in reporting and interpretation, we renamed the three latent factors as: (1) pregnancy with socioeconomic and well-being problems; (2) pregnancy with physical and mental health problems; and (3) pregnancy with maternal and fetal life-threatening conditions.

**Table 4:** Factor loading matrix after varimax rotation

Conditions	Factor 1	Factor 2	Factor 3	Uniqueness
1. To save a woman’s life	-0.1496	0.4687	***0.6023***	0.3952
2. To preserve a woman's mental health	0.1533	***0.8104***	0.0296	0.3189
3. To preserve a woman's physical health	0.0229	***0.7808***	0.2127	0.3445
4. Pregnant woman living with HIV/AIDS infection	***0.6376***	0.1815	-0.1059	0.5493
5. Failed contraception	***0.7319***	0.1230	-0.0293	0.4484
6. Pregnancy after rape	0.1226	***0.4612***	0.4319	0.5856
7. Pregnancy in incestuous relationship	***0.6381***	0.0607	0.1421	0.5690
8. Pregnancy in women aged below 15 years old	***0.7610***	0.0743	-0.0398	0.4138
9. Pregnancy in women aged 15–20 years old	***0.8713***	0.0619	0.0037	0.2370
10. Suffering from violence in family	***0.7656***	0.0374	0.1323	0.3949
11. Non-marital pregnancy	***0.8170***	-0.0389	-0.0698	0.3262
12. Socioeconomic problems	***0.7974***	0.0555	0.1336	0.3432
13. Fetal impairment	0.0168	0.1115	***0.8476***	0.2688
14. Abortion upon reasonable request after adequate counseling	***0.4608***	-0.0212	0.4728	0.5637

The bold and italic entries refer to factors that appear to be related to each other

### Determining the relationship between mode of admission and factor score

Table 5 shows the findings from univariate analysis. The graduates from the normal track were more agreeable to performing abortions than CPIRD doctors under the conditions of pregnancy with socioeconomic and well-being problems (factor score = 0.159 in normal track vs. − 0.142 in CPIRD) and pregnancy with physical and mental health problems (factor score = 0.092 in normal track vs − 0.082 in CPIRD) with a statistical significance (P-value < 0.001). In terms of pregnancy with maternal and fetal life- threatening conditions, the factor score in normal track graduates was − 0.012 whereas the same score for CPIRD graduates equated 0.011. However, there was no enough evidence demonstrating a difference between the two groups (P-value = 0.718).

**Table 5:** Findings from univariate analysis (t-test) of factor scores between CPIRD and normal track

Condition group—mean (SD)	Normal track	CPIRD	Differences (95% CI)	P-value
1. Pregnancy with socioeconomic and well-being problems	0.159 (1.085)	− 0.142 (0.903)	0.301 (0.173, 0.429)	< 0.001
2. Pregnancy with physical and mental health problems	0.092 (0.947)	− 0.082 (1.052)	0.174 (0.455, 0.304)	0.008
3. Pregnancy with maternal and fetal life-threatening conditions	− 0.012 (0.916)	0.011 (1.102)	− 0.024 (− 0.156, 0.107)	0.718

*SD* standard deviation, *95% CI* 95% confidence interval, *CPIRD* the Collaborative Project to Increase Production of Rural Doctors

Table 6 reveals factors associated with attitudes towards safe abortions in three different conditions. Males tended to be more agreeable than females to performing abortions for pregnant women with socioeconomic and well-being problems. Graduates from the central region were less agreeable to perform abortion services for women facing physical and mental health problems. The respondents with high confidence levels of ANC and the CPIRD graduates reported a lesser degree of agreement to provide abortion services under the reason related to socioeconomic and well-being problems (compared with those with low confidence levels of ANC and those admitted through the normal track). The respondents intending to become an OB-GYN doctor and the normal track graduates were more likely to have a positive attitude towards abortion in conditions of pregnancy with physical and mental health problems. For conditions of pregnancy with maternal and fetal life-threatening, none of the independent variables (including the type of admission) demonstrated a statistically significant relationship. For region variable, by using the northern region as a reference, no clear pattern on the association between regional domicile and abortion attitudes was observed. Participants originating from the southern part of Thailand showed a relatively positive attitude towards abortion services for mothers with physical and mental health problems. The respondents from the central region reported a relatively positive attitude in the conditions related to maternal and fetal life-threating. Nonetheless, the reverse pattern was found in the conditions involved pregnancy with physical and mental health problems.

**Table 6:** Findings from multivariable analysis of factor scores between CPIRD and normal track

Independent variables	Pregnancy with socioeconomic and well-being problems	Pregnancy with physical and mental health problems	Pregnancy with maternal and fetal life-threatening conditions
	Coeff^ξ^ (95%CI)	Coeff^ξ^ (95%CI)	Coeff^ξ^ (95%CI)
Age group equating 25 years or above (vs. age group below 25 years)	0.126 (− 0.025, 0.277)	− 0.089 (− 0.242, 0.063)	− 0.0123 (− 0.168, 0.143)
Male (vs. female)	0.198** (0.067, 0.329)	− 0.049 (− 0.182, 0.084)	− 0.051 (− 0.186, 0.084)
Region (vs. Northern)			
Central	0.051 (− 0.125, 0.227)	0.191* (0.013, 0.369)	− 0.253** (− 0.433, − 0.072)
Northeastern	− 0.071 (− 0.261, 0.118)	− 0.015 (− 0.207, 0.177)	− 0.028 (− 0.223, 0.167)
Southern	− 0.107 (− 0.306, 0.093)	0.208* (0.006, 0.410)	0.028 (− 0.177, 0.232)
CPIRD (vs. normal track)	− 0.226** (− 0.366, − 0.086)	− 0.118 (− 0.260, − 0.024)	0.055 (− 0.199, 0.089)
High confidence in providing ANC (vs. low confidence)	− 0.155* (− 0.301, − 0.008)	0.047 (− 0.101, 0.196)	− 0.137 (− 0.287, 0.014)
Intention to become an OB-GYN doctor (vs. no intention)	− 0.020 (− 0.306, 0.266)	0.344* (0.055, 0.634)	0.141 (− 0.153, 0.435)

ξ = coefficient; *refers to P-value < 0.05; **refers to P-value < 0.01

*CPIRD* the Collaborative Project to Increase Production of Rural Doctors, *ANC* antenatal care, *OB-GYN doctor* obstetrics and gynecologists

## Discussion

### Result discussion

This article is among the very first studies that explore attitudes of among new medical graduates in Thailand, about the conditions under which it is considered acceptable to perform safe abortions. Moreover, it is the first study that explores the difference in abortion attitudes between normal track graduates and CPIRD graduates in Thailand. We hope that findings from this study will be helpful for academic and policy makers in the field to revise the curricula for both normal track and CPIRD students and better prepare the working environment for new medical graduates to perform safe abortions in suitable conditions.

The most important reasons for which the respondents in this study agreed to perform safe abortions are: (1) to save a woman’s life; (2) to help a pregnant woman after being raped; and (3) to help a pregnant woman with fetal impairment. This finding coincides with a study by Boonthai et al. that surveyed abortion attitudes among approximately 3000 physicians in 2003. Boonthai et al. reported that these three conditions were among the most important reasons for physicians to be comfortable with performing abortions [17]. A recent study by Sanitya et al. also pointed in the same direction [18].

On the other hand, participants in this study were less agreeable to providing abortion services in certain conditions, namely, non-marital pregnancy and pregnancy in children and teenagers (either aged below 15 years or aged 15–20 years). This observation is interesting as pregnancy in a girl aged less than 15 years of age is a criminal offence, and abortion is permitted by law [5]. This means that most of the respondents in this survey seemed to be uncomfortable with providing abortions even where the law allows them to do so. A prior study by O'Connor et al. also pointed to the same direction, suggesting that about one third of GPs in Ireland denied women medicines to terminate pregnancy under conditions permitted by UK law [25]. Apart from the Ireland example, there are other countries which are open for legal abortion services (such as Nepal, South Africa and Tunisia), but in practice, there were still variations in practice and the denial of abortion services was commonly noticed [26].

In contrast, more than half of this study’s respondents agreed to offer abortion services for pregnant women ‘upon reasonable request after adequate counseling’ despite the fact this condition is not specified in the latest abortion law. This phenomenon might happen due to numerous reasons. For example, each individual respondent interpreted this question differently as the term ‘request after adequate counseling’ encompasses a vast range of conditions, from life-threatening problems to non-medical conditions. This pitfall in the questionnaire design will be discussed later in the ‘methodological discussion’ subsection.

Note that a few studies from abroad suggested that most medical students were relatively pro-choice (denoting an acceptable view to offer abortion services after adequate counselling). A research piece in UK informed that about two thirds of the medical students were pro-choice. Another study in Malaysia demonstrated that medical students were in need of further training on both pre- and post-abortion counselling skills [27]. A survey on medical students in the US illuminated that most medical students intended to incorporate abortion procedure into their future practices [28].

Concerning the comparison between attitudes in normal track graduates and CPIRD graduates, the normal track physicians tended to be more agreeable than CPIRD physicians with providing abortion services in pregnancies with socioeconomic and well-being problems, and pregnancies with physical and mental health problems. This phenomenon might be explained by the fact that the faculties of normal track students tended to work in well-prepared environments (mostly in university hospitals), relative to the faculties of CPIRD students (mostly in MOPH hospitals). The contextual environment is not just equipment and medical supplies but it means the whole health service system, which encompasses clear clinical practice guideline, multidisciplinary supporting team support, and availability of proper referral system and consultation services for complicated cases. All of these factors make physicians more comfortable to deal with women requiring a termination of pregnancy. In this respect, abortion services in university hospitals are likely to be better systematized and are more ready to deal with pregnant women with any conditions (whether they are medically related problems or socioeconomic problems) [29]. A study of Lucchetti et al. indicated that doctors in rural hospitals were more conservative (less likely to provide abortion) than urban hospitals (which were comparable to tertiary-care or university hospitals in the Thai context). Yet, this explanation is still indicative. The CPIRD and the normal track in essence apply the same pedagogic curriculum certified by the TMC. However, it is possible that students in different medical schools may have different learning experience since each medical school is allowed to slightly adapt or revise the curriculum to suit its context as long as the core content is still in line with the TMC standards.

A study by Myran et al. pointed to the interaction between sex of providers and abortion view, by suggesting that, for male medical students, the strength of intention to provide abortion diminished when students envisioned progressing through training into future practice. However, this pattern was not observed for female students [30]. Another study by Tey et al. argued that, in Malaysia, medical students’ attitudes toward abortion were not significantly associated with sex, type of university, or ethnicity [27].

It is also understandable that new graduates who plan to become OB-GYN specialists in the future may be more agreeable to providing abortion service for women with physical and mental health problems as they are more skillful in the medical procedures related to termination of pregnancy than those who are not willing to. Interestingly, the respondents with a high confidence in ANC appeared to be less agreeable to provide terminations of pregnancy for women with socioeconomic problems. Our assumption is there might be latent characteristics embedded in the ANC confidence. For example, medical graduates who were more skillful in ANC might have a world view more direct to physical health. Therefore, a request for pregnancy beyond physical health concern would be prone to disagreement in the view of these providers.

As mentioned earlier, we intended to use region of domicile in the early years of life of the respondents as a proxy for religious belief. Since the southern part of Thailand is the main habitat for Muslim Thais, we expected that participants from Southern Thailand would show a more negative attitudes towards abortion in all conditions—in line with the report in much prior literature [31–33]. However, the results revealed no specific association pattern between regional domicile and abortion views. This might be because the regional domicile could not serve as a good surrogate for religious belief as expected. Another possible reason is the regional domicile in early years of life were less influential than other determinants developed in the later years of life, such as social norm or worldview of an individual. Further study, especially a qualitative one, is recommended to provide a sensible explanation why and how certain variables (sex, religious upholding, confidence in ANC skill, and surrounding culture) interact with the abortion perspectives among healthcare providers.

### Methodological discussion

The strengths of this study are the recruitment of a vast number of new medical graduates (both CPIRD and normal track doctors) to participate in the survey and the use of various statistical quantitative techniques (descriptive statistics, factor analysis, univariate analysis, and multivariable regression analysis) to analyse attitudes towards abortion. However, some limitations remain. First, as we attempted to make the questionnaire as concise as possible, some demographic variables were not included in the questionnaire, such as religion, economic status, legal knowledge, and abortion-related experiences. To answer these questions, an in-depth interview is more appropriate than an online survey since some questions are quite sensitive and require respondents’ confidentiality (religion and household’s economic status). Second, it was difficult to ensure that all respondents understood the questions similarly. For instance, the participants might understand the term ‘counselling’ differently. In actual medical practice, counselling may refer to a process to reach agreement between doctors and service users about service alternatives or may refer to the provision of information about risk and benefits of the treatment that is already selected by healthcare providers. Third, the respondents in this study were limited to the new graduates who attended the public workplace selection forum only. This means that we did not have data of new doctors who intend to start their working life in the private sector from the outset. However, this limitation might not overly undermine the generalizability power of this study as about four fifths of the new physicians in Thailand still intend to work in the public sector. Besides, we did not enroll experienced providers in the study, let alone the OB-GYN specialists. Thus, the generalizability of the finding is limited to new graduates only. The readers should be cautious about this point as the attitudes reflected while being a new graduate do not necessarily reflect the attitudes developed the real-world clinical practice This is because the actual practice and the attitudes developed afterward are always influenced by a number of factors, such as readiness of the equipment, staff supervision, influence from medical peers, demand for services (such as abortion rate in a province or in a region) and resource availability of each individual facility.Last, the nature of a cross-sectional study design hinders the ability to suggest a causal inference. Though, some variables (like age and sex) are not likely to create a reverse causality, the variable related to the inclination to become OB-GYN specialist requires a more thorough investigation—and this possibly necessitates a more complicated study design (such as repeated measures of attitudes in each physician cohort).

To address these limitations, we recommend further studies that explore this issue in more detail. A study that delves into the characteristics of medical curricula related to reproductive health in both CPIRD and normal tracks are worth exploring. An additional survey on the new graduates that captures the existing unobserved variables, like religious beliefs, social norm and prior experience of abortion, should be performed; and it should include qualitative component to explore why and how certain factors influence the incorporation of abortion practices. A cohort study among new medical graduates to assess whether attitudes towards abortion change over time should be considered. As abortion services require a multi-disciplinary approach, additional studies on the abortion-related attitudes of other healthcare professionals, apart from physicians, are of great value.

## Conclusion

This study has shed light on Thailand’s new medical graduates’ attitudes towards safe abortion under various conditions. It found that the degree of acceptance of abortion services among new medical graduates did not always coincide with the Thai abortion law. The new doctors in this study appeared to be less agreeable to performing abortions for pregnant women who have problems that go beyond health issues to socioeconomic and other factors, such as pregnancy after rape, socioeconomic problems, and pregnancy in children and adolescents. The less agreeable attitude was more obvious in CPIRD physicians than normal track physicians. This might be explained by the fact that CPIRD doctors were trained in non-university settings, which were likely to be less prepared for abortion services than the university hospitals (the main study venues for normal track students). This point may lead to certain policy implications including the need for a more proactive campaign aimed at medical students, to raise awareness and mutual understandings of unintended pregnancy and safe abortion services. The CPIRD curricula relating to safe abortion should enhance the capacity of medical graduates to deal with pregnant women who face not only a physical health related problem, but also socioeconomic difficulties and well-being problem as a whole. To provide more suitable training for medical students, further studies should investigate the differences in learning experience between students in the the CPIRD and the normal track. A cohort study among new medical graduates to assess the dynamics of attitudes towards abortion services is worthwhile.

## Supplementary Information

**Additional file 1.** Intention to join each specialty training in medical graduates in 2018.

**Additional file 2.** Questions to assess attitudes for safe abortion services.

## Data Availability

The raw data used by this study jointly belonged to IHPP. The data are however available from the authors upon reasonable request.

## Abbreviations

ANC: Antenatal care
CPIRD: Collaborative Project to Increase Production of Rural Doctors
DOH: Department of Health
GP: General practitioner
MOPH: Ministry of Public Health
NGO: Non-government organization
OB-GYN: Obstetrics and gynecologists
PCF: Principal component factor
WHO: World Health Organization
