# Supplementary material for: Self-assessment of attitudes towards conditions to provide safe abortion among new medical graduates in Thailand, 2018: an application of cross-sectional survey with factor analysis
Source: BMC Womens Health. 2021 Jul 27;21:273. doi: 10.1186/s12905-021-01412-3 (PMC8314509; doi:10.1186/s12905-021-01412-3)
Supplement: Supplementary file 1 — Additional file 1. Intention to join each specialty training in medical graduates in 2018. [file 12905_2021_1412_MOESM1_ESM.docx]

|  | Specialty | No (%) |
| --- | --- | --- |
| 1 | Internal medicine | 139 (15.01) |
| 2 | Undetermined specialty | 120 (12.96) |
| 3 | Surgery | 116 (12.53) |
| 4 | Pediatric | 98 (10.58) |
| 5 | Preventive medicine | 81 (8.75) |
| 6 | Orthopedic | 60 (6.48) |
| 7 | OB-GYN | 49 (5.29) |
| 8 | Emergency medicine | 48 (5.18) |
| 9 | Psychiatry | 38 (4.10) |
| 10 | Ophthalmology | 33 (3.56) |
| 11 | Others | 144(15.56) |

**Table S1** Intention to join each specialty training in medical graduates in 2018
