# Supplementary material for: Self-assessment of attitudes towards conditions to provide safe abortion among new medical graduates in Thailand, 2018: an application of cross-sectional survey with factor analysis
Source: BMC Womens Health. 2021 Jul 27;21:273. doi: 10.1186/s12905-021-01412-3 (PMC8314509; doi:10.1186/s12905-021-01412-3)
Supplement: Supplementary file 2 — Additional file 2. Questions to assess attitudes for safe abortion services. [file 12905_2021_1412_MOESM2_ESM.docx]

**Table S2** Questions to assess attitudes for safe abortion services

**แบบสอบถามความรู้และทัศนคติต่อการให้บริการยุติการตั้งครรภ์ที่ปลอดภัย**

If you are a doctor who have to do abortion, which circumstance do you agreed

ถ้าท่านเป็นแพทย์ที่จะต้องให้บริการยุติการตั้งครรภ์ ท่านเห็นด้วยกับการให้บริการยุติการตั้งครรภ์ในกรณีใดต่อไปนี้บ้าง

| Circumstance | Extremely disagreed  (1) | Slightly disagreed  (2) | Neutral  (3) | Slightly agreed  (4) | Extremely agreed  (5) |
| --- | --- | --- | --- | --- | --- |
| 1. To save pregnant women’s life การกระทำเพื่อช่วยชีวิตหญิงตั้งครรภ์ |  |  |  |  |  |
| 1. Pregnant women have mental health problems ปัญหาสุขภาพทางจิตใจของหญิงตั้งครรภ์ |  |  |  |  |  |
| 1. Pregnant women have physical health problems ปัญหาสุขภาพทางกายของหญิงตั้งครรภ์ |  |  |  |  |  |
| 1. Pregnant women have HIV/AIDS หญิงตั้งครรภ์ติดเชื้อ HIV/AIDS |  |  |  |  |  |
| 1. Pregnancy from Failed contraception. หญิงตั้งครรภ์จากการคุมกำเนิดล้มเหลว (Failed contraception) |  |  |  |  |  |
| 1. Pregnancy from sex abuse e.g. rape หญิงตั้งครรภ์จากการถูกข่มขืนกระทำชำเรา หรือถูกล่อลวง |  |  |  |  |  |
| 1. Pregnancy from incest หญิงตั้งครรภ์กับญาติสายตรง |  |  |  |  |  |
| 1. Child’s pregnancy (less than 15 years old) การตั้งครรภ์ของวัยรุ่นอายุน้อยกว่า 15 ปีบริบูรณ์ |  |  |  |  |  |
| 1. Teenage pregnancy (15-19 years old) การตั้งครรภ์ของวัยรุ่นอายุ 15-19 ปี |  |  |  |  |  |
| 1. Pregnant women have family violence problems. หญิงตั้งครรภ์ที่มีปัญหาความรุนแรงในครอบครัว |  |  |  |  |  |
| 1. Pregnancy out of wedlock การตั้งครรภ์นอกสมรส |  |  |  |  |  |
| 1. Pregnant women have financial problems that hinder child raising. หญิงตั้งครรภ์มีปัญหาทางเศรษฐกิจไม่สามารถเลี้ยงดูบุตรได้ |  |  |  |  |  |
| 1. A fetal is disability or have seriously genetic diseases ตัวอ่อนในครรภ์มีความพิการหรือเป็นโรคทางพันธุกรรมรุนแรง |  |  |  |  |  |
| 1. Pregnant women want to terminate after counselling. หญิงตั้งครรภ์เลือกยุติการตั้งครรภ์ภายหลังได้รับการปรึกษาทางเลือก |  |  |  |  |  |
